# Hijacking or helping?—How political actors use the COVID-19 pandemic in the climate discourse to advocate their policy beliefs and preferences

**DOI:** 10.1007/s11077-025-09587-4

**Published:** 2025-09-08

**Authors:** Marlene Kammerer, Jack Baker, Lukas Paul Fesenfeld, Maiken Maier, Simon Montfort, Karin Ingold

**Affiliations:** 1 Institute of Political Science, University of Bern, Fabrikstrasse 8, 3012 Bern, Switzerland; 2 Oeschger Centre for Climate Change Research, University of Bern, Bern, Switzerland; 3 Centre for Comparative and International Studies, ETH Zurich, Zurich, Switzerland; 4 Environmental Social Science Department, Eawag, Dübendorf, Switzerland; 5 Laboratory on Human-Environment Relations in Urban Systems, EPF Lausanne, Lausanne, Switzerland; 6 Faculty of Social Sciences, University of Helsinki, Helsinki, Finland

**Keywords:** Climate policy, COVID-19 pandemic, Discourse analysis, Mixed-methods, Narrative policy framework

## Abstract

Many of today’s challenges, such as climate change, war, or health crises, are highly interlinked and intertwined. Actors in the public discourse sometimes use the term “polycrisis” to describe this “causal entanglement of crises”. This article investigates whether this entanglement is visible in the media discourse and whether political actors strategically (mis-)use simultaneous and overlapping crises to influence policymaking in favor of their policy beliefs and preferences. Specifically, it studies how the outbreak of the COVID-19 pandemic in 2020 influenced the climate discourse at that time and whether and how political actors included the pandemic as “narrative strategy” to advocate their climate policy beliefs and preferences. To answer this question, this article scrutinizes the climate media discourse in 2020 in Germany and Switzerland and employs a logistic regression model combined with a descriptive and qualitative analysis of the climate discourse in the two countries. Our results show that in both countries primarily pro-environment actors use COVID-19-related arguments as narrative strategy to increase public attention for the need of a more ambitious climate policy, while pro-economy actors follow a strategy of decreasing the salience of the climate issue (i.e., not linking the issues), potentially reducing public pressure for more ambitious climate mitigation.

## Introduction

Climate change is one of the most pressing challenges in today’s society, but it is not the only one, and many of today’s challenges are strongly interlinked and intertwined. In this context, the public political discourse, which is driven by international organizations, policymakers, and researchers, sometimes uses the term “polycrisis” to refer to the “causal entanglement of crises” (Lawrence et al., 2024). Specifically, the neologism has been used to describe the complex intertwinement of climate change, the war in Ukraine, and the COVID-19 pandemic (Tooze, 2022). However, is this close entanglement of different crises also visible in public political discourse? And do political actors strategically (mis)use the complexity of interactions between simultaneous and overlapping crises to advocate for their interests? Using the example of climate change and COVID-19, in this article, we aim to shed light on this question.

Due to the urgency of the problem and the emergence of movements like Fridays for Future, climate change has become one of the most salient issues in public political discourses in recent years (Gabehart et al., 2022; Kammerer & Ingold, 2023; Koteyko & Atanasova, 2016). But with the outbreak of the COVID-19 virus, the pandemic became a dominant topic in the media and challenged “the role of climate change as a routine issue” during this time (Eisenegger et al., 2020; Rauchfleisch et al., 2023). Despite the massive media attention to immediate problems linked to the COVID-19 pandemic (e.g., hospitalization or death rates, health and safety measures, etc.), the two topics were also discussed together (Rauchfleisch et al., 2023), often from a positive, pro-climate perspective that stressed the need to coordinate the paralleled crises (Stoddart et al., 2023). However, the pandemic was also used as an economic reason to reduce or pause climate mitigation ambition, i.e., as a contra-climate argument.^1^ In other words, political actors leveraged COVID-19-related arguments within the climate discourse as “narrative strategy” to advocate their climate policy beliefs and preferences (Jones et al., 2023).

Thus, the question arises of how the arrival of COVID-19 has affected the climate discourse and, more concretely, how actors in this discourse have dealt with and strategically used COVID-19-related arguments. Studying the effect of the start of the COVID-19 pandemic on climate discourse is particularly interesting and relevant, as COVID-19 became a highly polarized issue that cut across traditional pro-environment and pro-economy coalitions (Nachtwey et al., 2020). Hence, in this article, we ask how different political actors (e.g., governmental agencies, parties, civil society, businesses, or scientists) involved in climate discourse have used COVID-19-related arguments as a “narrative strategy” to push their own pro- or contra-climate mitigation policy beliefs and preferences. In doing so, we contribute to the literature on the ‘narrative strategies’ (Jones 2014; Jones et al., 2023) of political actors, showing how actors aligned with specific coalitions—as theorized in the Advocacy Coalition Framework (ACF)—draw on narratives to promote their beliefs in the face of simultaneous crises (Jones & McBeth, 2010).

Theoretically, we combine discourse analysis (Hajer & Versteeg, 2005) with the policy process literature. We present two hypotheses. First, we expect that actors involved in the climate discourse leverage COVID-19-related arguments in favor of their respective climate policy beliefs and preferences. Second, we assume that actors that support ambitious climate policy and challenge the status quo (typically actors with pro-environment beliefs and preferences) more frequently use COVID-19-related arguments as they seek to increase public attention than actors in favor of the status quo (typically actors with pro-economy beliefs and preferences) or such with more neutral positions.

We tested the hypotheses using a mixed method of quantitative and qualitative discourse analysis (Hajer, 1995; Hajer & Versteeg, 2005). In our analysis, we investigate Germany and Switzerland in 2020, the year the COVID-19 pandemic broke out. We analyzed the media discourse in these countries using eight key newspapers with large circulation rates across the left-to-right political spectrum. In total, we found over 10,000 potentially relevant articles in German and Swiss media discourses in 2020. Among these articles, we identified a total of 2647 climate-related statements made by diverse political actors. Next, we assessed whether these climate-related statements made direct reference to the COVID-19 pandemic. In other words, we assessed whether those COVID-19-related arguments were used as pro-climate (i.e., to advocate for more ambitious climate policy) or contra-climate (i.e., to advocate for the status quo or weaker climate policy) narrative strategy. We then used logistic regression to test our hypotheses and combined this with a brief descriptive and qualitative analysis of the COVID-19-related climate discourse.

In line with our first hypothesis, our results show that pro-economy actors use the COVID-19 pandemic to argue against a more ambitious climate policy and the maintenance of the status quo or even weaker climate policy, while pro-environment actors use the COVID-19 pandemic to argue for more ambitious climate mitigation. In addressing our second hypothesis, we find that political actors that typically pursue pro-economic beliefs and preferences and oppose strong mitigation measures (e.g., business interest groups, and conservative political parties) link the COVID-19 pandemic and climate change less often than the pro-environment coalition, which includes political actors who support environmental interests (e.g., left or green parties, pro-environment civil society organizations). In essence, pro-economy actors aim to decrease the salience of the climate change issue, potentially reducing public pressure to adopt more ambitious climate mitigation measures. In contrast, pro-environment actors use media attention around the COVID-19 pandemic to push their policy preferences for more stringent climate action. In this regard, we can draw conclusions about how simultaneous and sometimes competing crises can be (mis)used by political actors to promote their interests in other areas by developing relevant narrative strategies.

## Climate policy discourses

The media is a prominent arena of the policymaking process, where policy ideas, issues, and solutions are discussed (see Shanahan et al., 2008). For several decades, there has been increasing interest in the public policy literature (Leifeld, 2013) in the argumentative turn and the nexus between (media) discourses and policymaking (Fischer & Forester, 1993). In this context, policy discourse can be defined as a verbal interaction in mass or social media between actors about a certain public policy or element thereof (Bossner & Nagel, 2020; Kammerer & Ingold, 2023; Leifeld, 2014). In what follows, we first outline the intersection between discourse analysis, media, and policy processes before we develop hypotheses for our case.

### Policy discourses, media analysis, and policy process theories

Policy discourse analyses often study partial or entire policy processes and consider specific policy subsystems (Brandenberger et al., 2022; Fisher et al., 2013; Shanahan et al., 2011; Vogeler et al., 2021) given the notion that media and other platforms of “verbal interaction” are linked to and reflect at least certain aspects of policymaking (Leifeld, 2016; Schaub & Metz, 2020).

The related literature reveals a great interest in climate discourses. For example, scholarship has investigated the framing of climate change by the media (Boykoff, 2011; Broadbent et al., 2016; Schäfer & Schlichting, 2014), media impacts on public opinion (Brulle et al., 2012), political actors’ use of media to lay out their policy preferences (Boykoff, 2011), and the evolution of policy discourse over time (Kammerer & Ingold, 2023). A considerable number of climate discourse analyses adopt the Advocacy Coalition Framework (ACF) as a theoretical lens and focus on the identification of conflicting beliefs among the political actors about how to adapt to or mitigate climate change (Kammerer & Ingold, 2023). More concretely, these studies identify actors and their beliefs and policy preferences within one policy discourse to identify like-minded actors and aggregate them into opposing discourse coalitions. In this vein, Swarnakar et al. (2022) identified dominant actors and beliefs in India’s climate policy, whereas Kukkonen and Ylä-Anttila (2020) studied how scientific actors are coalition members in the Finnish climate policy discourse. Further, discourse coalitions, based on media data, tend to be skewed in favor of political actors advocating for a change of the status quo and less encompassing cover political-administrative actors (Schaub & Metz, 2020).

Overall, ACF applications and discourse analyses around the globe yield similar results (Gabehart et al., 2022), dividing the landscape of climate political actors in the following way: Political actors promoting ambitious climate action and policies, and thus offering “a solution to the climate problem,” are typically actors seeking to change the status quo and are thus part of the pro-environment coalition (Gronow et al., 2022; Kammerer & Ingold, 2023; Markard et al., 2022). Conversely, the pro-economy coalition with actors that support more economy-oriented interests and aim to either retain or weaken the status quo in climate policymaking; and an intermediary group of neutral actors who either seek a compromise between the pro-environment and pro-economy coalitions, change their preferences or introduce new ideas (e.g., clean-tech solutions) to the discourse (Gronow et al., 2022; Ingold, 2011; Markard et al., 2022; Tindall et al., 2015).

From the narrative policy framework (NPF), we have learned that actors try to influence policies through the stories they tell in political discourse and the media landscape (Swarnakar et al., 2022). The framework originated in the work of policy process scholars on the ACF (Jones & McBeth, 2010; McBeth & Shanahan, 2004; Shanahan et al., 2008, 2011), who concluded that (mass) media sources play an important role in advocacy coalitions’ diffusion of policy narratives, with embedded policy beliefs and strategies designed to promote a preferred policy outcome. Hence, narratives are of key strategic importance to the ability of political actors and advocacy coalitions—defined within the ACF by shared beliefs and policy preferences—to achieve their goals. Narrative strategies can thus be viewed as mechanisms through which coalitions externalize and advance their core beliefs in public discourse. For example, a recent application of the NPF to the promotion of agro-food technology policy in the European Parliament by Vogeler et al. (2021) shows that political actors advocate for their policy preferences based on narratives and argumentative reasoning.

In this vein, we divide actors involved in one specific policy subsystem or discourse (i.e., climate) into two main opposing coalitions and a more neutral group. We then further investigate whether these actors strategically use arguments from another policy issue or subsystem (i.e., the COVID-19 pandemic) to organize or manipulate their climate policy preferences (“narrative beliefs”; see Jones et al., 2023). In doing so, this research contributes to improving the understanding of the role of policy discourses in policymaking and how actors “behave” in overlapping or nested subsystems (Wiedemann & Ingold, 2022; Zafonte & Sabatier, 1998) in discourse perspective, which has been widely neglected so far (for an exception, see Boykoff, 2011; Brandenberger et al., 2022; Stoddart et al., 2023).

### The use of COVID-19 arguments in climate discourse: hypotheses

The many ACF applications using discourse analysis, which were introduced in "Policy discourses, media analysis, and policy process theories" section, demonstrate that actors involved in policy discourses outline their beliefs or policy preferences to advance their position, convince other actors, and, finally, influence the policy process and policy change in the preferred direction. Exogenous shocks that increase the salience of another subsystem issue, such as COVID-19, can also open new windows of opportunity for actors to promote their positions (Markard et al., 2022; Rinscheid et al., 2020). Accordingly, we expect actors in the climate discourse to use COVID-19-related arguments (i.e., frames related to the health subsystem) as what the NPF terms “narrative strategies” to reinforce their respective climate policy beliefs and preferences. Hence, we argue that pro-environment actors are more likely to draw COVID-19-related arguments as a narrative strategy in favor of ambitious climate policy, whereas pro-economy actors are likely to pursue the opposite strategy. For the neutral actors, we expect no common narrative strategy. We, therefore, hypothesize:

#### Hypothesis 1:

Political actors involved in climate discourse use COVID-19-related arguments to underpin their climate policy beliefs and preferences.

However, we assume that the two opposing coalitions and the neutral political actors pursue different narrative strategies. Specifically, we expect the pro-environment coalition to actively participate in the policy discourse and use COVID-19-related arguments as a narrative strategy in order to draw public attention to the need for more ambitious mitigation action and to influence policy change (Tosun & Schaub, 2017). In contrast, the pro-economic coalition, which would like to maintain the status quo, has less interest in increased public attention but instead might exploit more direct links to key decisionmakers (Fischer et al., 2017). Likewise, neutral actors have less incentive to increase public attention to the climate issue and, hence, participate less frequently in the climate discourse and are less likely to make use of COVID-related arguments. Accordingly, we hypothesize:

#### Hypothesis 2:

Pro-environment actors are more likely to use COVID-19-related arguments than neutral or pro-economy actors in the climate discourse.

## Research design

### Case study design

In this paper, our core research interest is to investigate the extent to which political actors involved in the climate discourse used the sudden start of the COVID-19 pandemic in 2020 to strategically employ arguments from the COVID-19 pandemic discourse to advocate for their climate policy beliefs and preferences. Here, we study the use of COVID-19-related arguments in climate discourse in two neighboring countries: Germany and Switzerland.

We selected Germany and Switzerland as case studies following a most-similar system design. Both countries are wealthy, democratic federal states with a longstanding environmental and climate discourse. They have both implemented climate mitigation acts with binding emission reduction targets, and while Germany employs a broader mix of climate policy instruments, previous studies have indicated that climate discourse in both countries is shaped by similar pro-environment and pro-economy coalitions (Kammerer et al., 2020a, 2020b).

Beyond climate policy, both countries experienced intense and polarized debates regarding their responses to the COVID-19 pandemic, making them suitable for an analysis of the pandemic’s impact on climate media discourse. The trajectory of the pandemic was comparable in Germany and Switzerland, with vaccination campaigns and lockdown measures fueling divisions that cut across traditional ideological and economic lines (Nachtwey et al., 2020. Specifically, in both countries, the issue) of COVID-19 created divisions that crossed through the pro-environment and pro-economy coalitions. For instance, many voters for the green parties in both countries, who have traditionally favored more ambitious climate and environmental policy, were divided about the question of vaccination and the role of the state in the context of the COVID-19 pandemic (Nachtwey et al., 2020).

Despite their similarities, some institutional and structural differences between the two cases should be mentioned. Switzerland’s direct democracy enables citizens to vote on climate policies, whereas Germany’s climate policy is embedded the COVID-19 pandemic in EU decision-making. Additionally, Switzerland’s smaller and less diverse media landscape contrasts with Germany’s larger and more fragmented media market. In this study, we can thus leverage the similarities between the countries while controlling for some institutional and structural differences in our analysis (see "Data analysis methods" section, "Regression models and results" section, and "Qualitative analysis" section).

Overall, our comparative most-similar case selection enhances internal validity by controlling for key structural similarities, allowing us to isolate the impact of the COVID-19 pandemic on climate discourse from unrelated economic or political differences. The nuanced institutional and media landscape variations in our cases introduce meaningful comparative leverage, enabling us to explore how political and media structures potentially moderate the impact of the COVID-19 pandemic on shifts in climate discourse. In terms of external validity, the findings are likely transferable to other wealthy Western European democracies with strong environmental movements. However, our design does not enable the generalization of results beyond this regional and political context—particularly to highly polarized media environments (e.g., the U.S.) or authoritarian states. This would require further comparative analyses beyond our two cases. Please refer to Appendix 1 for further details on the two cases.

### Data collection

To reconstruct the climate discourse during the COVID-19 pandemic in 2020, we analyzed media coverage from newspapers in Germany and Switzerland. This research forms part of a larger project investigating climate discourses in these countries between 2014 and 2021. The year 2021 was excluded from our analysis because only six relevant arguments concerning COVID-19 were made in that year across both countries.

The dataset was prepared through a three-step process following a standard procedure developed in the compon.org project (see Kammerer et al., 2021; Kammerer & Ingold, 2023). First, we collected a raw dataset of newspaper articles on climate policy from high-circulation media outlets that span the political spectrum. In Germany, these included *Süddeutsche Zeitung*, *Die Welt*, *Die Bild*, and *Die Tageszeitung*, while in Switzerland, we used *Tages-Anzeiger*, *Neue Zürcher Zeitung*, *Le Temps*, and *Blick*. Articles were retrieved using Factiva, a media database with extensive coverage, using German or French keywords such as “klima*,” “clima*,” or “CO2.” This search resulted in 6101 articles for Germany and 4106 for Switzerland.

Second, we pre-screened the articles to reduce irrelevant content while maintaining a comprehensive dataset. Articles were filtered based on keyword frequency (a minimum of three mentions) and length (a minimum of 250 words). This step excluded items like editorials, reader comments, and articles mentioning the keywords without a substantive focus on climate change. The pre-screening process, discussed in detail in Appendix 2, resulted in a refined corpus of 1547 articles for Germany and 1096 for Switzerland (see Table 1), suitable for manual annotation.

**Table 1:** Number of articles, statements, and actors

Workflow	Resulting articles	Germany	Switzerland
*Step 1*: Download articles from Factiva	All articles identified following search string	6101	4106
*Step 2*: Prescreening to exclude irrelevant articles before manual coding	Potentially relevant articles with keyword (e.g., climate, CO_2_) count ≥ 3 and article word count < 250	1547	1096
	Final corpus of articles containing actors and statement annotations (see below for more information)	438	399
	*Statements*		
	Total statements in the climate discourse COVID-19-related statements	1369 189	1278 75
*Step 3*: Manual annotation	*Actors*		
	Total actors	93	41
	Actors referring to COVID-19 (among total)	46	19

Third, we manually annotated the dataset using the Discourse Network Analyzer (DNA) software (Leifeld et al., 2018). This involved identifying and coding political actors—such as governmental agencies, political parties, civil society groups, and business actors—and their policy arguments. We analyzed different types of statements that reflect the beliefs and preferences of political actors. Policy statements were analyzed for narrative content, including statements on problem definitions (e.g., “climate change is real and anthropogenic”), policy targets (e.g., “drastic reduction in energy consumption”), or specific policy instruments (e.g., “preference for market-based instruments”). Statements were coded to reflect whether political actors agreed (“1”) or disagreed (“0”), which is a standard procedure in DNA applications. In the next step, these statements can be aggregated into more general beliefs and policy preferences and to identify opposing actor clusters. Regular coder meetings ensured consistency, and duplicate or overly specific statements were refined for comparability across countries. As displayed in Table 1, the final dataset included 93 political actors and 1369 policy statements for Germany and 41 political actors and 1278 policy statements for Switzerland.

The dataset, in its original form, is curated as a “classical” DNA dataset, the primary intention of which is to analyze the relations in a discourse network where actors are linked by shared beliefs and preferences, as described above. In this study, however, we use the dataset in a different way, as we focus on the statements themselves and how a subset of them is used in the discourse. More specifically, we were interested primarily in the statements that make specific COVID-19-related arguments within the climate discourse (see "Identification of COVID-19 related arguments in the climate discourse" section) and how and by whom they are used in the climate discourse.

### Identification of COVID-19 related arguments in the climate discourse

To identify how actors used arguments related to the COVID-19 pandemic as narrative strategies in the climate discourse, we identified different COVID-19-related arguments. We operationalized them as pro- or contra Covid-19-related arguments, as shown in Table 2.

**Table 2:** Categories of COVID-19-related arguments and their usage

COVID-19-related arguments	Argumentative usage	
	“Agreement”	“Disagreement”
Abolition/reduction of climate measures due to COVID-19 crisis	Contra-climate argument	Pro-climate argument
Tackling COVID-19 crisis should be coupled with CC mitigation	Pro-climate argument	Contra-climate argument
COVID-19 crisis presents opportunity for CC action	Pro-climate argument	Contra-climate argument
Need for more governmental support due to covid crisis (only Germany)	Contra-climate argument	Contra-climate argument

In the dataset, we identified four different argument categories. The first category (“Abolition/reduction of climate measures due to COVID-19 crisis”) argument category reflects actors opposing or seeking to reduce mitigation measures, often citing economic concerns. In some cases, actors explicitly disagreed with this view, emphasizing the importance of durable mitigation efforts. The second category (“Tackling covid crisis should be coupled with CC mitigation”) highlights calls for the integration of mitigation concerns into COVID-19 policies. For example, Svenja Schulze of the Social Democratic Party argued in the *Süddeutsche Zeitung* (April 28, 2020) for linking climate mitigation to be linked to stimulus programs, stating, “Future economic stimulus programs would have to be mandatorily geared towards climate mitigation. The methods and technologies for this are known” (own translation). The third category (“Covid crisis presents opportunity for CC action”) frames the pandemic as an opportunity for mitigation, often linked to shifts in travel habits or consumption patterns. Lastly, unique to Germany, some actors emphasized the need for increased governmental support to address the double burden of the two crises (“Need for more governmental support due to covid crisis”).

Further methodological details are provided in Appendix 2. For a list of political actors and statement categories, see Appendices 3 and 4 and Tables 8, 9, 10, 11, 12, 13 in Appendix 3 and 4.

### Data analysis methods

To test our hypotheses, we used a mixed-methods approach combining descriptive statistics, logistic regression, and qualitative analysis. While logistic regression was the main component of the analysis, both the descriptive statistics and qualitative analysis were intended to contextualize our findings.

For the logistic regression, we calculated two sets of models using two different dependent variables (DV), see Table 4 for an overview of all variables used in the regressions. In our first set of models (M1), our first dependent variable (DV 1) was whether COVID-19-related arguments, as presented in Table 2, were used as a pro-climate argument to advocate for more ambitious climate policy or as a contra-climate statement to advocate for weaker climate policy. As shown in Table 1, we identified 189 statements that included COVID-19-related arguments in Germany and 75 in Switzerland (i.e., a total of 264). Table 2 shows which statements we classified as pro-climate and which as contra-climate. In the second set of models (M2), DV 2 draws on the complete dataset (n = 2648); it is a dummy variable that indicates whether a statement includes a COVID-19-related argument (1) or not (0).

In both sets of models, our independent variable assigned the political actors to a typical *coalition* (see Appendix, Tables 8, 9). To avoid endogeneity issues, we did not determine the coalitions based on statement clusters in the policy discourse as often done in DNA studies (e.g., Kammerer & Ingold, 2023; Swarnakar et al., 2022). Instead, we identified them based on our expertise in the two cases (see Ingold, 2011; Kammerer & Ingold, 2023, Kammerer et al., 2020a). We categorized whether an actor is typically perceived as holding pro-economy, pro-environment, or neutral beliefs and preferences. The latter set of actors, often governmental agencies (see, for example, Ingold, 2011), cannot easily be allocated to one coalition or the other, as they sometimes hold pro-environment and sometimes proeconomy beliefs and preferences. We expected them not to draw on COVID-19-related arguments to advocate for their interests or to do so to a lesser degree. This resulted in a variable with three categories (i.e., *pro-environment*, *pro-economy*, and *neutral*). SeeTable 3 for the distriubtion COVID-19-related arguments across the three coalitions.

**Table 3:** Political actors by policy statements and COVID-19-related arguments

	Pro-environment	Pro-economy	Neutral	Total
Total statements	1419 (54%)	721 (27%)	502 (19%)	2647
COVID-19-related pro-climate arguments	136 (52%)	17 (6%)	34 (12%)	187
COVID-19-related contra-climate arguments	39 (17%)	23 (9%)	15(6%)	77
Total COVID-19-related arguments	175	40	49	264

Finally, we drew on several control variables for our analysis, as indicated in Table 4. First, we controlled for whether specific *actor types* that are usually involved in the climate policy subsystem use COVID-19-related arguments differently. We used that variable as an alternative measurement for the discourse coalition as described above. Specifically, we tested for civil society actors and business-related actors, as opposed to a mixed group of governmental actors, political parties, and science actors (Others).^2^ We assumed that civil society actors more often hold stances attributable to the proenvironment group, whereas the pro-business actors were presumably similar to those in the proeconomy coalition. Controlling for all actor types yields the same results. For a list of actors and their coding, please refer to Appendix 3, Tables 8 and 9.

**Table 4:** Overview of research design

Variable name	Description	Data source	Research design
COVID-19 pro- or contraargument	*Dummy variable*		
	1 = pro-argument 0 = contra-argument	Media discourse dataset	DV 1
COVID-19 argument	*Dummy variable*		
	1 = COVID-19 statement 0 = all others	Media discourse dataset	DV 2
Coalition	*Categorical variable*		
	Pro-environment Pro-economy Neutral	Own coding, based on literature	IV
Actor type	*Categorical variable*		
	Business (interest groups, companies) Civil society (NGOS, labor organizations) Others (government, political parties, science)	Own coding, based on literature	Control
Right-wing newspaper	*Dummy variable*		
	1 = Right-wing or conservative 0 = all others	Media discourse data set	Control
Country	*Dummy variable*		
	1 = Germany 0 = Switzerland	Media discourse data set	Control
COVID-19 wave	*Categorical variable*		
	1st wave (March to May) 2nd wave (from September) No wave	Schilling et al. (2021)	Control

To cover potential biases introduced by media outlets or the general tone of the discourse in the country, as well as during specific phases of the pandemic, we also controlled for the political orientation of the media outlet with *Right-wing newspaper*, *Country*, and the occurrence of *COVID-19 waves* in 2020. The first wave occurred from March to May 2020, and the second wave began in September 2020; see Schilling et al. (2021).^3^

For the qualitative analysis, we examined the identified COVID-19-related arguments and how they were used as pro- or contra-climate arguments (see Table 2) in greater detail by analyzing the individual argument categories. To this end, we read all the newspaper articles in which we identified COVID-19-related arguments. This step aimed to uncover the nuanced use of arguments and their context, complementing the quantitative analysis of the more aggregated coded COVID-19 statements (see Appendix 2 for details on the coding process). Specifically, we focused on how these general arguments were framed and the sectors in which they were applied (e.g., automobility or aviation). For example, actors opposing the argument of “abolishing or reducing climate crisis measures due to the COVID-19 crisis” framed their counterarguments by advocating for EU crisis recovery payments to prioritize sustainability criteria or by emphasizing the equal importance of addressing the climate crisis alongside the pandemic. This in-depth analysis of the four argument categories provides a richer understanding of the specific motives and strategies employed by different actors when utilizing COVID-19-related arguments within the climate discourse.

## Results

### Descriptive analysis

**Fig. 1:**
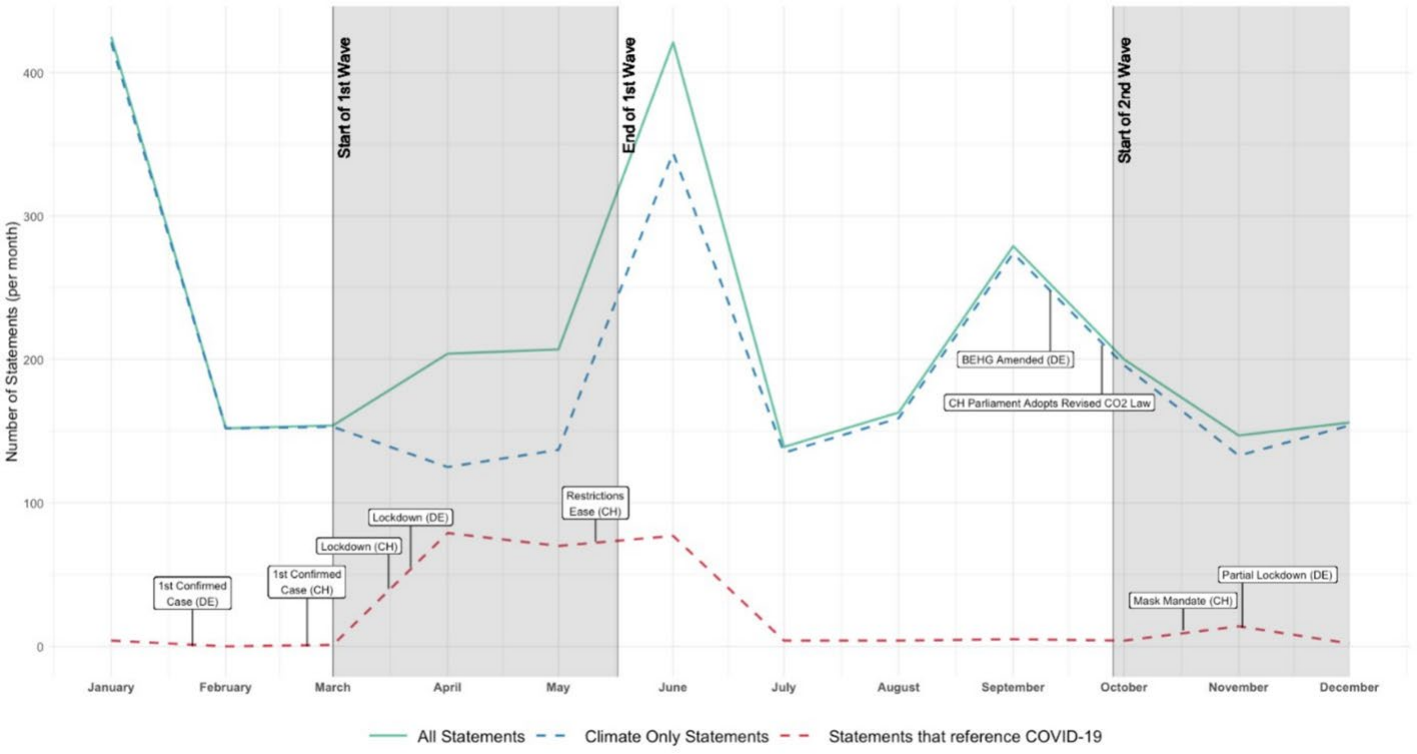
Development of statements in the climate discourse and COVID-19-related arguments in 2020

Aside from a small number of COVID-19-related comments in several articles in January 2020, presumably due to the outbreak of the novel virus at the end of the previous year, the climate discourses of Germany and Switzerland did not contain any COVID-19-related arguments (i.e., statements that referenced COVID-19 as an argument either pro- or contra-climate action). As shown in Fig. 1, there was a proliferation of COVID-19-related arguments during the first wave from April to June 2020.

In April, the number of COVID-19-related arguments reached its peak (79), and the number of statements that referred exclusively to climate change (i.e., with no reference to COVID-19) reached its lowest level for 2020. In May, the number of COVID-19-related arguments decreased, though only slightly. By June, the number of COVID-19-related arguments (77) increased to nearly peak levels. However, the number of statements that referred exclusively to climate change was much higher in June (344) than in April (125); thus, the proportion of all COVID-19-related arguments in June (2%) was much smaller than that in April (39%).

By July 2020, the overall number of COVID-19-related arguments declined, and climate discourses returned to pre-pandemic levels. During the portion of the second COVID-19 wave covered in this study (i.e., to the end of 2020), the overall number of COVID-19-related arguments slightly increased but did not return to the same heights seen during and immediately after the first wave. Overall, the climate discourses of Germany and Switzerland were most active (i.e., had the highest number of overall statements) in January, June, and September 2020, coinciding with international climate conferences held in December and June, which traditionally spur media attention toward the topic (Broadbent et al., 2016). Hence, during these times, the two crises competed for attention, and COVID-19-related arguments were often used as “narrative strategies” in the climate discourse in both countries. In September, there was a further peak in attention toward the climate discourse. At that time, in both countries, key climate policy-related events happened: in Germany, the Fuel Emissions Trading Act (BEHG) was amended, and in Switzerland, the parliament voted for the revised CO2-Act. Both events caused increased media attention. However, unlike during the first wave of the pandemic, COVID-19related arguments were rarely used.

Table 3 displays the political actors by coalition and the number of statements and COVID-19-related arguments. Of the 2647 statements made in the German and Swiss climate discourses in 2020. Overall, political actors in the pro-environment coalition were the most active, accounting for over half (54%) of all the statements made in 2020, followed by the pro-economy actors (27%) and the neutral actors (19%). Hence, it seems that pro-environment actors more actively participate in the climate discourse in order to increase public attention to the need for a more ambitious climate policy. Further, the table shows that political actors from each coalition made COVID-19-related arguments in the expected way, hence this provides first insights on our Hypothesis 1. However, it is less clear how pro-economy actors behave, as they make COVID-19-related arguments that are interpreted as pro-climate, hence, in almost half of the total COVID-19 related argument they make. The pro-environment actors in 39 instances use COVID-19-related arguments in a contra-climate way. Furthermore, actors from the pro-environment coalition used COVID-19-related arguments much more frequently as a narrative strategy than the pro-economy and neutral political actors. Finally, the neutral actors 49 times refer to COVID-19-related arguments in the climate discourse, in most instances in a pro-climate way. This allows several preliminary conclusions. Firstly, we find evidence for Hypothesis 1, but this is mostly driven by the fact that pro-environment actors very actively use the pandemic as a narrative strategy in the climate discourse. The case is far less clear for the pro-economy coalition. Second, and linked to that, we find evidence for the pro-environment actors to be more strongly participating in the discourse in general and also in terms of using COVID-19-related arguments (Hypotheses 2).

### Regression models and results

Tables 5 and 6 show the results of the logistic regression to test our two hypotheses. The models presented in Table 5 use a subset of statements with only COVID-19-related arguments, and the models presented in Table 6 use the full dataset containing all statements. The regression results clearly confirm both hypotheses.

**Table 5:** Results of logistic regression, Hypothesis 1, standard errors in parentheses

DV 1 = COVID-19 STATEMENTS AS ARGUMENTS	Model 1	Model 2	Model 3	Model 4	Model 5	Model 6	Model 7	Model 8	Model 9	Model 10	Model 11
INTERCEPT	0.89***	− 0.30	1.25***	− 0.99*	0.71**	− 0.09	1.69***	0.01	1.76***	0.43	1.80***
	(0.14)	(0.32)	(0.18)	(0.42)	(0.26)	(0.49)	(0.37)	(0.50)	(0.38)	(0.48)	(0.44)
PRO-ENVIRONMENT (BASE = PRO-ECONOMY)		1.55***		1.71***		1.78***		1.75***			
		(0.37)		(0.38)		(0.41)		(0.41)			
NEUTRAL (BASE = PRO-ECONOMY)		1.12*		1.27**		1.19*		1.18*			
		(0.45)		(0.46)		(0.47)		(0.48)			
PRO-ECONOMY (BASE = PRO-ENVIRONMENT)			− 1.55***		− 1.71***		− 1.78***		− 1.75***		
			(0.37)		(0.38)		(0.41)		(0.41)		
NEUTRAL (BASE = PRO-ENVIRONMENT)			− 0.43		− 0.44		− 0.59		− 0.57		
			(0.36)		(0.37)		(0.39)		(0.39)		
GERMANY				0.82**	0.82**	0.72*	0.72*	0.74*	0.74*	0.55	0.55
				(0.31)	(0.31)	(0.33)	(0.33)	(0.33)	(0.33)	(0.33)	(0.33)
FIRST WAVE						− 1.27***	− 1.27***	− 1.23***	− 1.23***	− 1.28***	− 1.28***
						(0.34)	(0.34)	(0.34)	(0.34)	(0.35)	(0.35)
SECOND WAVE						− 2.04***	− 2.04***−	2.01***	− 2.01***	− 1.91***	− 1.91***
						(0.52)	(0.52)	(0.52)	(0.52)	(0.51)	(0.51)
RIGHT-WING NEWSPAPER								− 0.30	− 0.30	− 0.23	− 0.23
								(0.31)	(0.31)	(0.31)	(0.31)
CIVIL SOCIETY (BASE = BUSINESS)										1.37**	
										(0.46)	
OTHERS (BASE = BUSINESS)										1.17**	
										(0.40)	
BUSINESS (BASE = CIVIL SOCIETY)											− 1.37**
											(0.46)
OTHERS (BASE = CIVIL SOCIETY)											− 0.20
											(0.39)
AIC	320.72	306.59	306.59	301.48	301.48	282.23	282.23	283.28	283.28	291.61	291.61
BIC	324.30	317.32	317.32	315.78	315.78	303.68	303.68	308.31	308.31	316.64	316.64
Log likelihood	− 159.36	− 150.29	− 150.29	− 146.74	− 146.74	− 135.11	− 135.11	− 134.64	− 134.64	− 138.80	− 138.80
Deviance	318.72	300.59	300.59	293.48	293.48	270.23	270.23	269.28	269.28	277.61	277.61
Num. obs.	264	264	264	264	264	264	264	264	264	264	264

*p*-values: 0.5 = *, 0.01 = **, 0.001 = ***

**Table 6:** Results of logistic regression, Hypothesis 2, standard errors in parentheses

DV 2 = COVID-RELATED ARGUMENT	Model 1	Model 2	Model 3	Model 4	Model 5	Model 6	Model 7	Model 8	Model 9	Model 10	Model 11
INTERCEPT	− 2.20***	− 2.83***	− 1.96***	− 3.53***	− 2.55***	− 4.02***	− 2.91***	− 3.98***	− 2.88***	− 3.46***	− 2.61***
	(0.06)	(0.16)	(0.08)	(0.20)	(0.13)	(0.23)	(0.14)	(0.23)	(0.15)	(0.23)	(0.18)
PRO-ENVIRONMENT (BASELINE = PRO-ECONOMY)		0.87***		0.98***		1.12***		1.10***			
		(0.18)		(0.18)		(0.19)		(0.19)			
NEUTRAL (BASELINE = PRO-ECONOMY)		0.60**		0.69**		0.75**		0.75**			
		(0.22)		(0.22)		(0.23)		(0.23)			
PRO-ECONOMY (BASELINE = PRO-ENVIRONMENT)			− 0.87***		− 0.98***		− 1.12***		− 1.10***		
			(0.18)		(0.18)		(0.19)		(0.19)		
NEUTRAL (BASELINE = PRO-ENVIRONMENT)			− 0.28		− 0.29		− 0.37*		− 0.36*		
			(0.17)		(0.17)		(0.18)		(0.18)		
COUNTRY (BASELINE = GERMANY)				1.01***	1.01***	1.14***	1.14***	1.14***	1.14***	0.95***	0.95***
				(0.14)	(0.14)	(0.15)	(0.15)	(0.15)	(0.15)	(0.15)	(0.15)
FIRST WAVE						1.45***	1.45***	1.46***	1.46***	1.50***	1.50***
						(0.15)	(0.15)	(0.15)	(0.15)	(0.15)	(0.15)
SECOND WAVE						− 0.66**	− 0.66**	− 0.66**	− 0.66**	− 0.69**	− 0.69**
						(0.24)	(0.24)	(0.24)	(0.24)	(0.24)	(0.24)
RIGHT-WING NEWSPAPER								− 0.10	− 0.10	− 0.10	− 0.10
								(0.14)	(0.14)	(0.15)	(0.15)
CIVIL SOCIETY (BASELINE = BUSINESS)										0.85***	
										(0.21)	
OTHERS (BASELINE = BUSINESS)										0.27	
										(0.20)	
BUSINESS (BASELINE = CIVIL SOCIETY)											− 0.85***
											(0.21)
OTHERS (BASELINE = CIVIL SOCIETY)											− 0.59***
											(0.17)
AIC	1720.12	1697.38	1697.38	1645.17	1645.17	1523.95	1523.95	1525.49	1525.49	1549.17	1544.61
BIC	1726.00	1715.02	1715.02	1668.70	1668.70	1559.24	1559.24	1566.66	1566.66	1590.34	1585.78
Log likelihood	− 859.06	− 845.69	− 845.69	− 818.59	− 818.59	− 755.98	− 755.98	− 755.75	− 755.75	− 767.58	− 765.30
Deviance	1718.12	1691.38	1691.38	1637.17	1637.17	1511.95	1511.95	1511.49	1511.49	1535.17	1530.61
Num. obs.	2648	2648	2648	2648	2648	2648	2648	2648	2648	2648	2648

*p*-values: 0.5 = *, 0.01 = **, 0.001 = ***

To test Hypothesis 1, we used the subset of the climate discourse that only contains COVID-19-related arguments. The dependent variable indicates whether a specific COVID-19-related argument was used as a pro (1) or contra argument (see also Table 2). The results show that actors typically associated with the pro-environment coalition were 5.75 times more likely to use pro-climate COVID-19-related arguments than actors from the pro-economy coalition or 1.7 times more likely than the neutral actors. The neutral set of actors also used COVID-19-related arguments 3.25 more likely to advocate for more ambitious climate policy (i.e., “pro-climate”) than the pro-economy actors, but not more likely than the pro-environment actors.

The models presented in Table 6 support the second hypothesis. Here, we used as a dependent variable all COVID-19-related arguments (1) as opposed to all other statements (0). As hypothesized, in the overall climate discourse, we see that pro-environment actors generally use COVID-19-related arguments 2.4 times more likely than pro-economy actors and the neutral set of actors.

Our results also remained robust when adding further variables to the models for both hypotheses. Overall, COVID-19-related arguments were used more often in German climate discourse than in Swiss discourse and were also used more often as pro-climate arguments (see Models 5.4 to 5.11 and 6.4 to 6.11). Further, the use of COVID-19 arguments during the infection waves differed, as they were more likely to be used as pro-climate arguments during the first wave and as contra-climate arguments in the second wave (see Models 5.6 and 5.11). There was no significant difference in political orientation across the different media outlets (see Models 5.8 to 5.11 and 6.8 to 6.11).

In Models 5.10, 5.11,6.10, and 6.11, we tested for political actor type. Civil society actors were more likely to use COVID-19-related arguments as pro-climate arguments. The opposite holds for business actors, who more often used COVID-19-related arguments in a contra-climate fashion. Furthermore, the results show that civil society actors, who are typically associated with pro-environment preferences (see Table 2), were more likely to use COVID-19-related arguments in the climate discourse. Conversely, business actors were less likely to use COVID-19-related arguments. These results are unsurprising and provide a robustness check for the expert-based operationalization of the coalition variable.

The DV has an imbalanced distribution in both datasets (indicated by the significant intercept parameter), meaning that there are more instances of COVID-19-related arguments used to support proclimate than contra-climate beliefs and preferences and far fewer COVID-19-related-arguments than regular statements with no reference to COVID-19. To ensure that the imbalance did not bias our results in favor of the majority group, we ran robustness checks with upsampled data. Fortunately, the results remained robust (see Appendices Table 12 for further details).

### Qualitative analysis

A qualitative assessment of the COVID-19-related arguments identified in climate discourse in Switzerland and Germany (see "Data analysis methods" section) demonstrates that a large portion of COVID-19-related arguments in the discourse discussed whether the response to the COVID-19 pandemic should be coupled with climate mitigation, for example, in the form of sustainability criteria that must be fulfilled to qualify for certain crisis recovery aid packages.

In line with the first hypothesis, we see that actors involved in the climate discourse use COVID-19-related arguments in favor of their pro- or contra-climate policy preferences, respectively. More specifically, pro-environment actors argue in favor of coupling the COVID-19 pandemic with climate mitigation measures, whereas pro-economy actors oppose such proposals, given the changed and uncertain economic situation. For instance, some pro-economy actors in Germany, including business actors and the German Free Democratic Party, even demand the abolition or reduction of climate targets already in place, for example, in the airline and car industries, due to the COVID-19 situation. Although the Swiss Liberal Party and the Swiss People’s Party also argue for abolishing or reducing climate targets, this is not as prominent in the Swiss discourse overall. In contrast, pro-environment actors in both countries stress that the importance of tackling climate change remains unchanged, stating that it should be of equally high priority as addressing the COVID-19 pandemic.

The analysis so far has shown that it is primarily pro-environment actors that leverage COVID-19-related arguments. Thus, we qualitatively analyzed what types of arguments pro-environment actors used when combining COVID-19 and the climate discourse. For instance, pro-environment actors in the Swiss discourse, including scientific actors and the Green Party Switzerland, distinctively compared the two crises to highlight their similarities and differences and to emphasize that the climate crisis should be addressed with at least the same urgency and determination as the COVID-19 pandemic. Moreover, scientific actors in the Swiss discourse stressed that climate change exacerbates the risk of future pandemics, highlighting the need for stricter policy measures. In contrast, one unique line of reasoning in the German discourse included some pro-environment actors (e.g., civil society actors and the Green Party), stating that the government should reduce the mitigation obligations to help companies during the COVID-19 pandemic. Yet, while acknowledging the need for some COVID-19-related measures to assist the economy, these pro-environment actors still argued in favor of maintaining climate targets. Furthermore, scientific actors in both countries framed the COVID-19 pandemic as a chance to sustainably transform the economy and society, arguing that the COVID-19 pandemic was a unique opportunity to advance climate policy. For instance, the National Academy of Sciences Leopoldina in Germany described the crisis as “a unique opportunity to overcome the COVID-19 pandemic and move the entire economy in a new, forward-looking, green direction” (own translation; *Die Welt*, April 16, 2020). Lastly, pro-environment actors in Germany and Switzerland, including some scientific actors, civil society groups, and political parties, argued for better climate policies, warning that the COVID-19 pandemic had relegated climate discourse to the background, which may have led to an exacerbation of the climate crisis. As a representative from the Swiss Climate Delegation stated, “There is a risk that emissions will rise further as the economy seeks to recoup its losses and people return to a mood of consumption and flying after the crisis” (own translation; *Tages Anzeiger*, April 5, 2020).

## Discussion and conclusions

In this paper, we analyzed how actors involved in climate discourse used COVID-19 arguments as a narrative strategy to leverage their climate policy beliefs and interests. To do so, we systematically coded newspaper articles related to climate change in Germany and Switzerland during the first two years of the pandemic, from 2020 to 2021, and adopted a mixed-methods approach to test our hypotheses.

We draw on the ACF and the NPF by theorizing that political actors form coalitions that jointly advocate for their policy beliefs and preferences by using similar narrative strategies (Hypothesis 1). While proenvironment actors leveraged the pandemic to advocate for stronger climate action, framing the crises as interconnected challenges requiring coordinated responses, pro-economy actors largely avoided linking the two crises, likely in an effort to reduce the pressure for more ambitious mitigation measures. Related to the second hypothesis, our results further show that pro-environment actors in both countries leveraged COVID-19 more frequently than pro-economy or neutral actors did. In general, these findings provide evidence that when facing overlapping crises, actors selectively frame issues to either amplify or diminish their political salience. In this regard, this paper contributes to the current discussion of how and when policy issues span across the boundaries of subsystems (Brandenberger et al., 2022; Wiedemann & Ingold, 2022).

Further, this study highlights the utility of combining discourse analysis with policy process theories to understand how crises are used as political instruments. Our analysis contributes to an understanding of how the ACF and NPF can complement each other: actors embedded in belief-based coalitions, as conceptualized in the ACF, use narrative strategies, as developed in the NPF, to shape discourse and influence policy outcomes. In this way, narrative strategies operationalize coalition beliefs in discursive arenas. Or put differently, actors use narrative strategies, including the thematic framing of an “outsider” policy problem, to underline their beliefs and policy preferences in one specific policy subsystem or discourse. This is interesting because it shows how issues from another subsystem (such as health) can be used narratively in an existing policy subsystem or debate (such as the climate).

By situating our findings within the concept of polycrisis, we contribute to a deeper understanding of how multiple crises interact within political discourses. The entanglement of the COVID-19 pandemic and climate change is not just a coincidence of timing, but a reflection of how political narratives evolve in response to competing crises. Our results suggest that actors who advocate for transformative change are more likely to exploit emergent crises as windows of opportunity, whereas those defending the status quo may strategically work to decouple issues with the aim of maintaining existing power structures.

The findings also raise important questions for future research, particularly regarding how different political systems and institutional settings shape the effectiveness of crisis-driven policy narratives. Further comparative studies across diverse governance contexts and longer timeframes would help refine the understanding of how polycrisis dynamics influence policy trajectories and why certain crises generate lasting policy shifts while others fade quickly from discourse.

Interestingly, we found almost exclusively COVID-19-related arguments in the 2020 climate discourse (except for a small number of arguments in 2021). This is an indication that using the COVID-19 pandemic as a basis for a narrative strategy was short-lived, occurring primarily during the first wave of the COVID-19 pandemic. As the pandemic progressed, the climate discourse largely returned to prepandemic patterns, where political actors stopped to use the pandemic as a narrative strategy. This suggests that while crises can serve as political windows of opportunity, their impact on discourse is often transient, particularly when other pressing issues dominate the public and the political agenda.

Our study and approach also contain some shortcomings and limitations. More countries and cases are needed to sufficiently facilitate an empirical basis from which conclusions can be drawn regarding the role of institutional settings, politics, and the political system in overlapping subsystems. A useful future research avenue for additional comparative case studies could be to apply a most-different system design, examining countries with different institutional, political, and economic structures and levels of polarization to further assess the robustness and generalizability of our findings. Furthermore, it would be crucial to incorporate the temporal dimension into future work, that is, to consider the time preceding and following the crisis, in addition to “during-crisis” scenarios, to conclude how policy preferences develop in complex and overlapping settings. Lastly, various studies have highlighted the role of media reporting biases in news articles in general across news outlets (Dallmann et al., 2015; Hamborg et al., 2019) and in relation to the COVID-19 pandemic in particular (Eisenegger et al., 2020; Starosta et al., 2020). This reporting bias may have impacted the results of the present study. However, studies on media biases have also shown that media reporting can have a strong and significant impact on public perception of a topic (Hamborg et al., 2019), making the analysis of COVID-19-related arguments in the climate discourse very relevant.

In conclusion, our results are not only valuable from theoretical and empirical perspectives but are also of practical relevance. It is interesting to know that actors presenting solutions to a problem, rather than promoting the status quo, seem to better leverage a new issue on the media agenda to support their preferences. Often, the status quo coalition dominates decision-making and makes change in a subsystem difficult. Therefore, minority coalitions may make more use of a new issue than dominant coalitions as a strategy to overcome their dominance. In practical terms, these insights are valuable for policymakers, activists, and media strategists seeking to navigate the increasingly complex landscape of crisis-driven policymaking. Recognizing the ways in which crises are selectively framed—and understanding how these framings are used as narrative strategies—can provide a more nuanced perspective on policy inertia and change, helping to anticipate how future crises—whether economic, geopolitical, or environmental—might be strategically leveraged within political discourse.

## Appendix 1: Detailed case descriptions

### Germany: Climate policymaking

Climate policymaking in Germany dates to the 1980s. With the start of the Green party, a strong pro-environment coalition emerged that since its start continuously engaged in a discursive conflict with a status-quo-oriented pro-economy coalition. The emergence of the strong pro-environment coalition in the 1980s was one of the main reasons, why Germany became one of the first countries to establish a comprehensive climate policy with the adoption of the *Climate Protection Program* (Klimaschutzprogramm) (von Hirschhausen, 2018). In 2005, the German government introduced the *Renewable Energy Sources Act* (Erneuerbare-Energien-Gesetz, EEG), which established a feed-in tariff system to promote the development of renewable energy sources. The EEG has been credited with helping to make Germany a leader in renewable energy and globally reducing the costs of renewable energy production (Brunner, 2008; Meckling & Nahm, 2018; Pahle et al., 2018; Schmidt & Sewerin, 2017; Schmidt et al., 2019). In the past years, Germany has faced criticism for continuing to rely on coal for a significant portion of its energy needs and not meeting its emissions reduction targets (Climate Action Tracker 2023). In reaction to the criticism, the government has taken steps to address these issues, including setting a target to phase out coal by 2038 and increasing support for renewable energy. Yet, protests continue given the lack of progress in reducing emissions, for example in the transport sector. Furthermore, as a reaction to the salient public protests led by the Fridays for Future movement and public concern over the impacts of climate change, the *Climate Protection Act* (Klimaschutzgesetz) was adopted in 2019 as a comprehensive and binding framework for achieving the country’s emissions reduction targets. In 2021, the emission reduction targets set in the *Climate Protection Act* were revised after a ruling of the Constitutional Court found them to be insufficient.

### Germany: The COVID-19 pandemic in 2020

The first COVID-19 case in Germany was confirmed on January 27, 2020. In the following weeks, the number of cases in Germany began to increase, with the first cases of community transmission reported in late February. The German government responded quickly to the outbreak, implementing a range of measures to slow the spread of the virus. Despite these measures, the number of cases in Germany continued to rise, peaking in late March and early April. The country was able to flatten the curve through a combination of social distancing measures, widespread testing and contact tracing, and a well-equipped healthcare system. As cases began to decline in the summer of 2020, Germany gradually lifted many of its restrictions. However, a second wave of the pandemic hit the country in the fall, leading to a new round of lockdowns and restrictions on public life. In December 2020, Germany began a vaccination campaign, with priority given to healthcare workers and the elderly. However, the rollout was initially slow, and there were concerns about vaccine supply shortages. Especially, the vaccination campaign led to polarization, cutting across traditional pro-economy and pro-environment coalitions.

### Switzerland: Climate policymaking

Switzerland’s climate policy has a long tradition. The first attempts to introduce a carbon tax date back to the 1970s and were linked to the oil crises, which increased the awareness of the need to reduce the dependency on imported fossil fuels. While Switzerland has always been a pusher of international climate mitigation measures, at home its climate policy can be characterized as a perpetual conflict between two opposing advocacy coalitions (Ingold, 2011; Kammerer & Ingold, 2023). On one side, actors from the economy, energy, and transport sectors, as well as right-wing parties make up the pro-economy coalition. On another side, the pro-environment coalition consists of civil society organizations, non-governmental organizations, and green and leftist parties. In contrast to Germany, in Switzerland, climate policymaking has centered around one core policy, the so-called Swiss CO_2_ Act which is comprised of various individual policy measures. The first version of the Swiss CO_2_ Act was weak and relied on voluntary measures. In 2008 a carbon tax on combustibles from industry was introduced, but a tax on motor fuels was blocked, either by a majority in Parliament or at the ballot by the people. In June 2023, the Swiss voters finally voted in favor of the Climate and Innovation Act revised CO_2_ Act that should help Switzerland comply with the promises made under the Paris Agreement. This revised policy is focused more strongly on innovation-oriented policies rather than market-based disincentives, like emission pricing (Kammerer et al., 2023).

### Switzerland: The COVID-19 pandemic in 2020

The COVID-19 pandemic reached Switzerland in late February 2020, when the country reported its first case. The first wave of the pandemic hit Switzerland hard, and the number of cases and deaths rose sharply in March and April. The Swiss government took a series of measures to contain the spread of the virus, including closing schools, banning large gatherings, and implementing social distancing guidelines. In May, the government began easing restrictions, and by June, most businesses and schools had reopened. However, cases began to rise again in July. The second wave of the pandemic hit Switzerland in October, and cases and deaths surged once again. In response, the government implemented stricter measures, including a nationwide mask mandate in all indoor public spaces, a ban on gatherings of more than five people, and the closure of bars and restaurants. Similar to Germany, also Switzerland started to vaccinate, first elderly and weak segments of society, in December 2020. Also, in Switzerland the vaccination issue led to a polarized public debate.

## Appendix 2: Optimizing the trade-off between sample comprehensiveness and manual coding effort for high-quality manual annotation

As described in the main article, we used an article exclusion approach based on keyword-matching and word count to filter out articles that are unlikely to contain relevant actors and statements. Specifically, we excluded articles that contained our keywords (“klima*”, “clima*”, and “CO_2_”) less than three times and were shorter than 250 words to yield a comprehensive sample of articles. This procedure optimises a trade-off between comprehensiveness and manual coding effort.

To ensure that this trade-off is optimized at a useful point, we first compute the omission rate. The omission rate measures the share of relevant articles that are falsely omitted when applying the above-described exclusion approach to our keywords. We estimate the omission rate by randomly sampling 75 articles from each set of excluded articles in Germany and Switzerland. We checked manually if these articles contained actors and statements. To calculate the omission rate, we simply divide the number of articles in the random sample of excluded articles that are falsely excluded, i.e. contain actors and statements, by the total number of the random sample (i.e., 75)

$$Omission\,rate = \frac{Number\,of\,articles\,with\,statements\,by\,actors\,in\,the\,random\,sample}{{Total\,number\,of\,articles\,in\,the\,random\,sample}}$$

In Germany, 12% of the articles in the random sample were excluded but could have been annotated with a statement and an actor; in Switzerland, it was 21% (see Table 7).

**Table 7:** Numbers for the computation of the omission and coding rates

Country	Numbers for the omission rate			Numbers for the coding rate		
	Number of articles with statements by actors in the random sample of excluded articles	Total number of articles in the random sample of excluded articles	Omission rate	Number of articles with actors and statements after exclusion	Total number of articles after exclusion	Coding rate
Germany	9	75	0.12	438	1547	0.28
Switzerland	16	75	0.21	399	1096	0.36

Next, we determine the coding rate. The coding rate is the share of articles with annotations on actors and statements among the sample of articles after applying the exclusion approach. That is, the coding rate is calculated by dividing the number of articles annotated with actors and statements by the total number of articles in the sample after applying the exclusion approach.

$$Coding\;rate = \frac{Number\;of\;articles\;with\;actors\;and\;statements\;after\;exclusion}{{Total\;number\;of\;articles\;after\;exclusion}}$$

For Germany, 28% of all manually screened articles were annotated and contained a statement by an actor; for Switzerland, it was 36% (see Table 7). Lastly, the comparison between the coding rate and the omission rate in Table 7 shows that the restricted sample selected for manual coding obtained through the keyword filtering approach contained more relevant articles than the sample we omitted: For Germany, the coding rate is more than twice as high as the omission rate and for Switzerland, it is more than 1.5 times as high. In summary, we deem this point of the trade-off between comprehensiveness and relevance of articles suitable to limit the coding effort while maintaining high-quality manual annotation at a manageable level.

## Appendix 3: Actor’s list

See Tables 8 and 9.

**Table 8:** Swiss political actors involved in the climate-COVID-19 policy discourse, their aliases, types, and the cumulative frequency of their statements in the discourse

Policy actor name	Policy actor alias	Policy actor type	Frequency
Financial industry	FIN	Business	82
Corporations	CORP	Business	35
Insurance/pension fund	INSUR	Business	16
Trade association	SGV	Business	16
Energy industry	ENRGY	Business	14
Economiesuisse	ECON	Business	12
Airline industry	AIR	Business	11
Consulting	CONSULT	Business	11
Agro industry	AGRO	Business	10
Car importers	CAR	Business	10
Swisscleantech	CLEANTECH	Business	7
Traffic organization	TRAF	Business	4
Houseowner's association	HEV	Business	3
Tourism	TOUR	Business	2
Climate activist	ACT	Civil Society	94
NGOs	NGO	Civil Society	42
WWF Switzerland	WWFS	Civil Society	17
Gold Standard	GOLD	Civil Society	12
Greenpeace Schweiz	GREEN	Civil Society	12
Federal government	GOV	Government	61
Parliament	PARL	Government	61
Federal Office of Environment	FOEN	Government	24
Environmental Commission of Parliament	ESPEC	Government	11
Federal Office of Energy	SFOE	Government	8
Financial Market Supervisory	FINMA	Government	6
Environmental department	DETEC	Government	5
Financial department	FDF	Government	4
Financial Commission of Parliament	FC	Government	3
Agency for Development and Cooperation	DEZA	Government	2
Judge	JUDGE	Government	1
Secretariat for Economic Affairs	SECO	Government	1
World Economic Forum	WEF	IGO	4
Green Party Switzerland	GPS	Political Party	138
Swiss People's Party	SPP	Political Party	107
Free Democratic Party	FDP	Political Party	74
Socialdemocratic Party	SPS	Political Party	65
Greenliberal Party	GLP	Political Party	42
Die Mitte	MITTE	Political Party	35
Evangelical People's Party	EPP	Political Party	3
Research institution	ACADEMIA	Science	213

**Table 9:** German political actors involved in the climate-COVID-19 policy discourse, their aliases, types, and the cumulative frequency of their statements in the discourse

Policy actor name	Policy actor alias	Policy actor type	Frequency
Energy industry	ENERGY	Business	37
Agro industry	AGRO	Business	30
NGO	NGO	Business	25
Agora energy transition	AGORA	Business	22
Consulting	CONSULT	Business	20
Siemens Inc	SIE	Business	18
Corporation	CORP	Business	17
Other organization	OTHER	Business	12
Salzgitter	SG	Business	12
Volkswagen group	VW	Business	12
Association of the Automotive industry	AAIA	Business	11
Verdi	VERDI	Business	10
The Federation of German Consumer Organizations	VZBV	Business	9
Air industry	AIR	Business	7
Generations Foundation	GF	Business	7
Vattenfall	VAT	Business	7
German Chamber of Industry and Commerce	IHK	Business	6
E.on	E.ON	Business	6
Shell	SHELL	Business	6
BMW Inc	BMW	Business	5
German Farmers' Union	DBV	Business	5
Fraunhofer Society	FS	Business	5
IKEA	IKEA	Business	5
Uniper	UNIPER	Business	5
Association of Municipal Enterprises	AME	Business	5
Vonovia	VON	Business	5
Federal Wind Energy Association	BWE	Business	4
Federal Association of German Industry	BDI	Business	4
Federal Association of German Housing and Real Estate Enterprise Registered Associations	BDWI	Business	4
Federal Association of German Waste Management, Water and Raw Materials Management	BDEWR	Business	4
German Energy Agency	DEnA	Business	4
German Hydrogen and Fuel Cell Association	GHFCA	Business	4
Evonik	EVK	Business	4
Kering	KER	Business	4
Thyssenkrupp	THY	Business	4
Association of German Mechanical and Plant Engineering	AGMPE	Business	4
Consumer Organization	CONS	Business	3
Construction Industry	CONSTR	Business	2
Financial industry	FI	Business	2
Insurance/Pension Fund	INSUR	Business	2
Tourism industry	TOURISM	Business	1
Fridays for Future	FFF	Civil Society	129
Research Institution	ACADEMIA	Civil Society	60
Greenpeace	GREEN	Civil Society	39
German Federation for the Environment and Nature Conservation	BUND	Civil Society	30
Ende Gelände	EndeGelände	Civil Society	25
Extinction Rebellion	XR	Civil Society	19
Transport industry	TRANS	Civil Society	19
Environmental Action Germany	EAG	Civil Society	18
Nature and Biodiversity Conservation Union	NABU	Civil Society	15
Climate Activist	ACTIV	Civil Society	13
Interest Union Mining, Chemicals, Energy	IGBCE	Civil Society	8
Urgewald	URG	Civil Society	8
WWF Germany	WWF	Civil Society	8
Covestro	CovStro	Civil Society	7
Germanwatch	GerW	Civil Society	7
All Villages Stay	ADB	Civil Society	6
Plant-for-the-Planet	PFTP	Civil Society	6
Stay on the Ground	STAY	Civil Society	4
Client Earth	CE	Civil Society	4
German Naturschutzring	GNR	Civil Society	4
E3G	E3G	Civil Society	4
Interest Union Metal	IUM	Civil Society	4
Citizens	Citizens	Civil Society	2
Relief Organization	RELIEF	Civil Society	1
Federal Government	Breg	Government	39
Federal Ministry for Economic Affairs and Energy	BMWi	Government	34
Federal Ministry for the Environment, Nature Conservation, Nuclear Safety and Consumer Protection	BMUV	Government	28
Advisory Council for Environmental Issues	SVRU	Government	8
Federal Ministry of Transport	BMV	Government	7
European Union	EU	Government	7
German Federal Ministry of Research and Technology	BMBF	Government	5
Federal Environment Agency	UBA	Government	5
The Federal Ministry of Transport and Digital Infrastructure	BMDV	Government	1
Federal Association of Energy and Water Management	BMDV	Government	1
Alliance 90 / The Greens	GREENS	Political Party	149
Social Democratic Party of Germany	SDP	Political Party	57
Christian Democratic Union	CDU	Political Party	45
The Left	LEFT	Political Party	41
Free Democratic Party	FDP	Political Party	33
Christian Social Union	CSU	Political Party	14
Alternative for Germany	AFD	Political Party	7
Research institution	ACADEMIA	Science	89

## Appendix 4: List of policy statements

See Tables 10 and 11.

**Table 10:** List of policy statement categories and their frequency of the concepts in the Swiss discourse (COVID-19-related statements in bold)

Statement	Frequency
Revision of the CO2 Act	104
Activism is a legitimate way to tackle CC	84
Air ticket levy	57
Ways of consumption should be changed to mitigate CC	52
Climate neutrality	40
National measures in aviation sector needed	40
CC as risk	39
Companies are important for climate protection	39
CC is currently occurring, and its impacts are visible	37
**Tackling covid crisis should be coupled with CC mitigation**	37
**Covid crisis presents opportunity for CC action**	34
Green investments	34
Expansion of renewable energy	33
My country’s (federal) climate protection ambition / measures sufficient	33
Urge for immediate action (no wait-and-see strategy)	32
Climate action creates jobs and opportunities for economic growth	31
CO2 tax	27
Low emission (private / public) transport	26
National reduction target 50%	25
Domestic climate action first	23
Divest from fossil fuels	21
Market-based instruments instead of regulation	21
Promotion of fossil energy	19
International coordination	17
Stricter regulation of vehicles	17
Subsidies (for GHG reduction)	17
CC mitigation should not endanger economic competitiveness	15
Climate Cent valuable contribution to reduce emissions	15
Voluntary instruments	15
Financial sector is not aligned with the goals of the Paris Agreement	14
Great(est) potential for GHG reductions/energy savings in the building sector	14
Gletscherinitiative	13
CC action politically feasible	12
Increased CO2 tax on combustibles	12
Intergenerational justice	12
Central banks should play an active role in climate protection	11
Climate research is important for promoting actions	10
Energy supply can be secured exclusively by renewable energies	9
Individual responsibility for climate protection action	9
My country should take a leading international role in GHG reduction	9
Swiss Climate Fund	9
Positive view of current climate negotiations	8
Promotion of technical solutions	8
Redistribution of CO2 tax	8
CC is one of biggest challenges of humanity	7
Coal phase-out	6
Energy law or strategy	6
Expanding or sustaining nuclear energy	6
Greater transparency of banks' climate-intensive investments needed	6
Clean Development Mechanism	5
Climate awareness is low	5
Coordination with EU CC regime	5
Current US Administration is a development for climate protection	5
Emission trading	5
My country is particularly vulnerable to CC	5
My country is responsible for small % of global emissions	5
Prevention of CC risks necessary	5
**Abolition/ reduction of climate measures due to covid crisis**	4
Ban on fossil fuels needed	4
CCS technology	4
Capitalism puts CC mitigation at risk	4
Human activities are a major driver of CC	4
International carbon tax	4
My country should be an international leader in climate finance	4
Wealth tax for climate protection	4
Financial and technology transfer to developing countries	3
Preventing greenwashing	3
Reforestation and avoided deforestation strategies	3
Developed countries should bear the main responsibility in GHG reductions	2
Earmarking of CO2 tax	2
Increasing energy efficiency	2
Large-scale power plants essential for energy security	2
Off-roader Initiative	2
2000-Watt-Society	1
A strong, binding international agreement is necessary	1
Compensation of fuel imports	1
Each country should be able to decide own reduction target (principle of self-determination)	1
Green New Deal	1
Grey emissions should be counted	1
Higher standard for CO_2_ certificates	1
International GCC agreement needs to include all major emitters	1
Municipals/cities important for CC	1
My country should ratify the Paris Agreement	1
Reduction of other GHGs	1
Significant carbon sink potential in soil	1

**Table 11:** List of policy statement categories and their frequency of the concepts in the German discourse (COVID-19 related statements in bold)

Statement	Frequency
**Tackling covid crisis should be coupled with CC mitigation**	126
Expansion of renewable energy	118
My country’s (federal) climate protection ambition / measures sufficient	116
Low emission (private / public) transport	111
Ways of consumption or production should be changed to mitigate CC	88
Coal phase-out	86
Activism is a legitimate way to tackle CC	47
**Covid crisis presents opportunity for CC action**	45
CC is currently occurring, and its impacts are visible	43
CC mitigation should not endanger economic competitiveness	34
Promotion of fossil energy	32
Climate action creates jobs and opportunities for economic growth	25
Climate neutrality	25
Promotion of technical solutions	25
Environmental tax	24
Reforestation and avoided deforestation strategies	24
CO2 tax	22
Energy law or strategy	22
**Abolition/reduction of climate measures due to covid crisis**	21
Climate research is important for promoting actions	21
Companies are important for climate protection	21
Market-based instruments instead of regulation	21
Intergenerational justice	20
Capitalism endangers CC mitigation	17
**Need for more governmental support due to covid crisis (e.g., EEG)**	16
CC as risk	15
Great(est) GHG reduction potential in a specific sector	15
Urge for immediate action (no wait-and-see strategy)	15
Climate justice	13
Green investments	13
Subsidy (GHG reduction)	13
My country should take a leading international role in GHG reduction	12
National measures in aviation sector needed	12
CC is one of biggest challenges of humanity	11
International coordination	9
Planning security for energy transition	9
Increasing energy efficiency	8
National reduction targets	8
CO2 label for food	7
Emission trading	7
Significant carbon sink potential in soil	7
CCS technology	6
Expanding or sustaining nuclear energy	6
CC action politically feasible	5
Coordination with EU CC regime	5
Prevention of CC risks necessary	5
Stricter regulation of vehicles	5
Earmarking of CO_2_ tax	4
Gas-fired power plants	4
Individual responsibility for climate protection action	4
Lowering administrative hurdles for the energy transition	4
My country is responsible for small % of global emissions	3
Remuneration for ecosystem services	3
Climate awareness is low	2
National climate budget	2
Voluntary instruments	2
Inclusion of carbon sequestration of forests into climate regime	1
Naming and shaming tactic	1
Safe and affordable energy supply	1

## Appendix 5: Robustness checks

See Tables 12 and 13.

**Table 12:** Results logistic regression using upsampled data, Hypothesis 1, standard errors in parentheses

DV 1 = COVID-19 STATEMENTS AS AGRUMENTS	Model 1	Model 2	Model 3	Model 4
INTERCEPT	0.79**	− 0.68	− 0.70	0.76*
	(0.27)	(0.41)	(0.39)	(0.34)
PRO-ECONOMY (BASELINE = PRO-ENVIRONMENT)	− 1.48***			
	(0.34)			
NEUTRAL (BASELINE = PRO-ENVIRONMENT)	− 0.76**			
	(0.29)			
COUNTRY (BASELINE = GERMANY)	0.78**	0.78**	0.72**	0.72**
	(0.26)	(0.26)	(0.26)	(0.26)
FIRST WAVE (BASELINE = NO WAVE)	− 1.10***	− 1.10***	− 1.24***	− 1.24***
	(0.25)	(0.25)	(0.26)	(0.26)
SECOND WAVE (BASELINE = NO WAVE)	− 1.88***	− 1.88***	− 1.79***	− 1.79***
	(0.41)	(0.41)	(0.41)	(0.41)
RIGHT-WING NEWSPAPER	− 0.43	− 0.43	− 0.13	− 0.13
	(0.24)	(0.24)	(0.24)	(0.24)
PRO-ENVIRONMENT (BASELINE = PRO-ECONOMY)		1.48***		
		(0.34)		
NEUTRAL (BASELINE = PRO-ECONOMY)		0.71		
		(0.39)		
CIVIL SOCIETY (BASELINE = BUSINESS)			1.46***	
			(0.36)	
OTHERS (BASELINE = BUSINESS)			1.25***	
			(0.33)	
BUSINESS (BASELINE = CIVIL SOCIETY)				− 1.46***
				(0.36)
OTHERS (BASELINE = CIVIL SOCIETY)				− 0.21*
				(0.29)
AIC	455.81	455.81	459.74	459.74
BIC	483.28	483.28	487.21	487.21
Log likelihood	− 220.91	− 220.91	− 222.87	− 222.87
Deviance	441.81	441.81	445.74	445.74
Num. obs.	374	374	374	374

*p*-values: 0.5 = *, 0.01 = **, 0.001 = ***

**Table 13:** Results logistic regression using upsampled data, Hypothesis 2, standard errors in parentheses

DV 2 = COVID-RELATED STATEMENT	Model 1	Model 2	Model 3	Model 4
INTERCEPT	− 0.77***	− 1.95***	− 1.37***	− 0.49***
	(0.07)	(0.10)	(0.10)	(0.08)
PRO-ECONOMY (BASELINE = PRO-ENVIRONMENT)	− 1.17***			
	(0.09)			
NEUTRAL (BASELINE = PRO-ENVIRONMENT)	− 0.37***			
	(0.09)			
COUNTRY (BASELINE = GERMANY)	1.30***	1.30***	1.08***	1.08***
	(0.07)	(0.07)	(0.07)	(0.07)
FIRST WAVE (BASELINE = NO WAVE)	1.60***	1.60***	1.56***	1.56***
	(0.08)	(0.08)	(0.08)	(0.08)
SECOND WAVE (BASELINE = NO WAVE)	− 0.70***	− 0.70***	− 0.66***	− 0.66***
	(0.10)	(0.10)	(0.09)	(0.09)
RIGHT-WING NEWSPAPER	− 0.14*	− 0.14*	− 0.12	− 0.12
	(0.07)	(0.07)	(0.07)	(0.07)
PRO-ENVIRONMENT (BASELINE = PRO-ECONOMY)		1.17***		
		(0.09)		
NEUTRAL (BASELINE = PRO-ECONOMY)		0.80***		
		(0.11)		
CIVIL SOCIETY (BASELINE = BUSINESS)			0.89***	
			(0.10)	
OTHERS (BASELINE = BUSINESS)			0.30***	
			(0.09)	
BUSINESS (BASELINE = CIVIL SOCIETY)				− 0.89***
				(0.10)
OTHERS (BASELINE = CIVIL SOCIETY)				− 0.59***
				(0.08)
AIC	5578.96	5578.96	5714.79	5714.79
BIC	5624.24	5624.24	5760.07	5760.07
Log likelihood	− 2782.48	− 2782.48	− 2850.39	− 2850.39
Deviance	5564.96	5564.96	5700.79	5700.79
Num. obs.	4768	4768	4768	4768

*p*-values: 0.5 = *, 0.01 = **, 0.001 = ***

## Footnotes

1 See for example: https://www.un.org/en/desa/no-pandemic-did-not-help-climate-action.

2 We aggregated these actors to reduce the number of categories in the regression.

3 The beginning and end of the COVID-19 waves in Switzerland (Wolfisberg et al., 2021) differed by a matter of days, up to a week at most, from those in Germany (Schilling et al., 2021). Given that this difference is marginal, we used the German dates for both countries. Moreover, as the second wave lasted until February 2021, it was not analyzed in its entirety in this study.

## References

[CR1] Bossner, F. R., & Nagel, M. (2020). Discourse networks and dual screening: Analyzing roles, content and motivations in political Twitter conversations. *Politics and Governance,**8*(2), 2020. 10.17645/pag.v8i2.2573

[CR2] Boykoff, M. T. (2011). *Who speaks for the climate?: Making sense of media reporting on climate change*. Cambridge University Press.

[CR3] Brandenberger, L., Ingold, K., Fischer, M., Schläpfer, I., & Leifeld, P. (2022). Boundary spanning through engagement of policy actors in multiple issues. *Policy Studies Journal,**50*(1), 35–64. 10.1111/psj.12404

[CR4] Broadbent, J., Sonnett, J., Botetzagias, I., Carson, M., Carvalho, A., Chien, Y.-J., Edling, C., Fisher, D., Giouzepas, G., Haluza-DeLay, R., Hasegawa, K., Hirschi, C., Horta, A., Ikeda, K., Jin, J., Ku, D., Lahsen, M., Lee, H.-C., Lin, T.-L.A., & Zhengyi, S. (2016). Conflicting climate change frames in a global field of media discourse. *Socius: Sociological Research for a Dynamic World,**2*(3), 237802311667066. 10.1177/2378023116670660

[CR5] Brulle, R. J., Carmichael, J., & Jenkins, J. C. (2012). Shifting public opinion on climate change: An empirical assessment of factors influencing concern over climate change in the U.S., 2002–2010. *Climatic Change,**114*(2), 169–188. 10.1007/s10584-012-0403-y

[CR6] Brunner, S. (2008). Understanding policy change: Multiple streams and emissions trading in Germany. *Global Environmental Change,**18*(3), 501–507. 10.1016/j.gloenvcha.2008.05.003

[CR8] Dallmann, A., Lemmerich, F., Zoller, D., & Hotho, A. (2015). Media bias in German Online newspapers. In *Proceedings of the 26th ACM Conference on Hypertext & Social Media—HT ’15* (pp. 133–137). 10.1145/2700171.2791057

[CR9] Eisenegger, M., Oehmer, F., Udris, L., & Vogler, D. (2020). *Die Qualität der Medienberichterstattung zur Corona-Pandemie* (p. 24). fög—Forschungszentrum Öffentlichkeit und Gesellschaft—Universität Zürich. 10.5167/uzh-196619

[CR10] Fischer, F., & Forester, J. (1993). *The argumentative turn in policy analysis and planning*. Duke University Press.

[CR11] Fischer, M., Angst, M., & Maag, S. (2017). Co-participation in the Swiss Water Forum network. *International Journal of Water Resources Development,**2*(1), 1–19.

[CR12] Fisher, D. R., Leifeld, P., & Iwaki, Y. (2013). Mapping the ideological networks of American climate politics. *Climatic Change,**116*(3–4), 523–545. 10.1007/s10584-012-0512-7

[CR13] Gabehart, K. M., Nam, A., & Weible, C. M. (2022). Lessons from the advocacy coalition framework for climate change policy and politics. *Climate Action,**1*(1), 1. 10.1007/s44168-022-00014-5

[CR14] Gronow, A., Satoh, K., Ylä-Anttila, T., & Weible, C. M. (2022). Of devils, angels and brokers: How social network positions affect misperceptions of political influence. *Journal of European Public Policy*. 10.1080/13501763.2022.2046137

[CR15] Hajer, M. (1995). *The politics of environmental discourse: Ecological modernization and the policy process*. Clarendon Press.

[CR16] Hajer, M., & Versteeg, W. (2005). A decade of discourse analysis of environmental politics: Achievements, challenges, perspectives. *Journal of Environmental Policy & Planning,**7*(3), 175–184. 10.1080/15239080500339646

[CR17] Hamborg, F., Donnay, K., & Gipp, B. (2019). Automated identification of media bias in news articles: An interdisciplinary literature review. *International Journal on Digital Libraries,**20*(4), 391–415. 10.1007/s00799-018-0261-y

[CR19] Ingold, K. (2011). Network structures within policy processes: Coalitions, power, and brokerage in Swiss climate policy. *Policy Studies Journal,**39*(3), 435–459.

[CR23] Jones, M. D. (2014). Communicating climate change: Are stories better than “Just the Facts”? *Policy Studies Journal,**42*(4), 644–673. 10.1111/psj.12072

[CR24] Jones, M. D., & McBeth, M. K. (2010). A narrative policy framework: Clear enough to be wrong? *Policy Studies Journal,**38*(2), 329–353. 10.1111/j.1541-0072.2010.00364.x

[CR22] Jones, M. D., Smith-Walter, A., McBeth, M. K., & Shanahan, E. A. (2023). The narrative policy framework. In *Theories of the policy process* (5th ed.). Routledge.

[CR28] Kammerer, M., & Ingold, K. (2023). Actors and issues in climate change policy: The maturation of a policy discourse in the national and international context. *Social Networks,**75*, 65–77. 10.1016/j.socnet.2021.08.005

[CR25] Kammerer, M. Ingold. K. & Crameri, F. (2020a). Das Klima und die EU: Eine Diskursperspektive für die Deutsche und Schweizerische Klimapolitik. In P. Careja, P. Emmenegger, & N. Giger (Eds.), *The European social model unter pressure—Liber Amoricum in honor of Klaus Armingeon* (pp.599–623).

[CR30] Kammerer, M., Ingold, K., & Dupois, J. (2020b). Switzerland: International commitments and domestic drawbacks. In R. Wurzel & M. S. Andersen (Eds.), *Leading the way: Pioneers, leaders and followers in multi-level and polycentric climate governance. *Routledege.

[CR26] Kammerer, M., Satoh, K., Vesa, J., Howe, A., Baker, J., & Montfort, S. (2021). *COMPON DNA coding instructions version for Switzerland*. 10.13140/RG.2.2.32497.20324

[CR27] Kammerer, M., et al. (2023). Climate governance and federalism: Switzerland. In A. Fenna, S. Jodoin, & J. Setzer (Eds.), *Climate governance and federalism: A forum of federations comparative policy analysis. *Cambridge University Press.

[CR31] Koteyko, N., & Atanasova, D. (2016). Discourse analysis in climate change communication. In *Oxford Research Encyclopedia of climate science*. 10.1093/acrefore/9780190228620.013.489

[CR201] Kukkonen & Ylä-Anttila (2020). https://www.cogitatiopress.com/politicsandgovernance/article/view/2603

[CR32] Lawrence, M., Homer-Dixon, T., Janzwood, S., Rockstöm, J., Renn, O., & Donges, J. (2024). Global polycrisis: The causal mechanisms of crisis entanglement. *Global Sustainability,**7*, 1–16.

[CR34] Leifeld, P. (2013). Reconceptualizing major policy change in the Advocacy Coalition Framework: A discourse network analysis of German pension politics. *Policy Studies Journal,**41*(1), 169–198. 10.1111/psj.12007

[CR35] Leifeld, P. (2014). Polarization of coalitions in an agent-based model of political discourse. *Computational Social Networks,**1*(1), 7. 10.1186/s40649-014-0007-y

[CR36] Leifeld, P. (2016). Discourse network analysis: policy debates as dynamic networks. In J. N. Victor, A. H. Montgomery, M. Lubell, M. T. Heaney, & J. M. Strickland (Eds.), *The Oxford handbook of political networks. *Oxford University Press.

[CR33] Leifeld, P., Gruber, J., & Bossner, F. R. (2018). *Discourse network analyzer manual*. https://github.com/leifeld/dna/releases

[CR37] Markard, J., Rinscheid, A., & Widdel, L. (2022). Analyzing transitions through the lens of discourse networks: Coal phase-out in Germany. *Environmental Innovation and Societal Transitions,**40*, 315–331. 10.1016/j.eist.2021.08.001

[CR38] McBeth, M. K., & Shanahan, E. A. (2004). Public opinion for sale: The role of policy marketers in Greater Yellowstone policy conflict. *Policy Sciences,**37*(3), 319–338. 10.1007/s11077-005-8876-4

[CR39] Meckling, J., & Nahm, J. (2018). The power of process: State capacity and climate policy. *Governance,**31*(4), 741–757. 10.1111/gove.12338

[CR40] Nachtwey, O., Frei, N., & Schäfer, R. (2020). *Politische Soziologie der Corona-Proteste* [Working Paper]. Universität Basel. 10.31235/osf.io/zyp3f

[CR41] Pahle, M., et al. (2018). Sequencing to ratchet up climate policy stringency. *Nature Climate Change,**8*(10), 861–867. 10.1038/s41558-018-0287-6

[CR42] Rauchfleisch, A., Siegen, D., & Vogler, D. (2023). How COVID-19 displaced climate change: Mediated climate change activism and issue attention in the Swiss media and online sphere. *Environmental Communication,**17*(3), 313–321. 10.1080/17524032.2021.1990978

[CR43] Rinscheid, A., Eberlein, B., Emmenegger, P., & Schneider, V. (2020). Why do junctures become critical? Political discourse, agency, and joint belief shifts in comparative perspective. *Regulation & Governance,**14*(4), 653–673. 10.1111/rego.12238

[CR44] Schäfer, M. S., & Schlichting, I. (2014). Media representations of climate change: A meta-analysis of the research field. *Environmental Communication,**8*(2), 142–160. 10.1080/17524032.2014.914050

[CR45] Schaub, S., & Metz, F. (2020). Comparing discourse and policy network approaches: Evidence from water policy on micropollutants. *Politics and Governance,**8*(2), 184–199. 10.17645/pag.v8i2.2597

[CR46] Schilling, J., Tolksdorf, K., Marquis, A., Faber, M., Pfoch, T., Buda, S., Haas, W., Schuler, E., Altmann, D., Grote, U., Diercke, M., & RKI COVID-19 Study Group. (2021). Die verschiedenen Phasen der COVID-19-Pandemie in Deutschland: Eine deskriptive Analyse von Januar 2020 bis Februar 2021. *Bundesgesundheitsblatt - Gesundheitsforschung - Gesundheitsschutz,**64*(9), 1093–1106. 10.1007/s00103-021-03394-x 10.1007/s00103-021-03394-x PMC8353925 34374798

[CR47] Schmidt, T. S., Schmid, N., & Sewerin, S. (2019). Policy goals, partisanship and paradigmatic change in energy policy—Analyzing parliamentary discourse in Germany over 30 years. *Climate Policy,**19*(6), 771–786. 10.1080/14693062.2019.1594667

[CR48] Schmidt, T. S., & Sewerin, S. (2017). Technology as a driver of climate and energy politics. *Nature Energy*. 10.1038/nenergy.2017.84

[CR50] Shanahan, E. A., Jones, M. D., & McBeth, M. K. (2011). Policy narratives and policy processes. *Policy Studies Journal,**39*(3), 535–561. 10.1111/j.1541-0072.2011.00420.x

[CR52] Shanahan, E. A., McBeth, M. K., Hathaway, P. L., & Arnell, R. J. (2008). Conduit or contributor? The role of media in policy change theory. *Policy Sciences,**41*(2), 115–138. 10.1007/s11077-008-9058-y

[CR53] Starosta, K., Onete, C. B., Grosu, R., & Plesea, D. (2020). *COVID-19 mass media infodemic in six European countries* [Preprint]. 10.31124/advance.13333697.v1

[CR54] Stoddart, M. C. J., Ramos, H., Foster, K., & Ylä-Anttila, T. (2023). Competing crises? Media coverage and framing of climate change during the COVID-19 pandemic. *Environmental Communication,**17*(3), 276–292. 10.1080/17524032.2021.1969978

[CR55] Swarnakar, P., Shukla, R., & Broadbent, J. (2022). Beliefs and networks: Mapping the Indian Climate Policy Discourse surrounding the Paris Climate Change Conference in 2015. *Environmental Communication,**16*(2), 145–162. 10.1080/17524032.2021.1973528

[CR56] Tindall, D., Robinson, J., & Stoddart, M. C. J. (2015). A view from sociology: Environmental movement mobilization over old growth temperate rainforests in British Columbia. In S. M. Redpath, R. Gutiérrez, K. A. Wood, & J. C. Young (Eds.), *Conservation conflicts: Navigating towards solutions* (pp. 152–164). Cambridge University Press.

[CR57] Tooze, A. (2022). Defining polycrisis—From crisis pictures to the crisis matrix. https://adamtooze.substack.com/p/chartbook-130-defining-polycrisis

[CR58] Tosun, J., & Schaub, S. (2017). Mobilization in the European public sphere: The struggle over genetically modified organisms. *Review of Policy Research,**34*(3), 310–330.

[CR59] Vogeler, C. S., Schwindenhammer, S., Gonglach, D., & Bandelow, N. C. (2021). Agri-food technology politics: Exploring policy narratives in the European Parliament. *European Policy Analysis,**7*(S2), 324–343. 10.1002/epa2.1114

[CR60] von Hirschhausen, C. (Ed.). (2018). *Energiewende “Made in Germany.”* Springer International Publishing.

[CR61] Wiedemann, R., & Ingold, K. (2022). Solving cross-sectoral policy problems: Adding a cross-sectoral dimension to assess policy performance. *Journal of Environmental Policy & Planning,**24*(5), 526–539. 10.1080/1523908X.2021.1960809

[CR62] Wolfisberg, S., Gregoriano, C., Struja, T., Kutz, A., Koch, D., Bernasconi, L., Hammerer-Lercher, A., Mohr, C., Haubitz, S., Conen, A., Fux, C. A., Mueller, B., & Schuetz, P. (2021). Comparison of characteristics, predictors and outcomes between the first and second COVID-19 waves in a tertiary care centre in Switzerland: An observational analysis. *Swiss Medical Weekly,**151*(3132), w20569. 10.4414/smw.2021.20569 34375985 10.4414/smw.2021.20569

[CR63] Zafonte, M., & Sabatier, P. (1998). Shared beliefs and imposed interdependencies as determinants of ally networks in overlapping subsystems. *Journal of Theoretical Politics,**10*(4), 473–505. 10.1177/0951692898010004005

